# Predictors of reoperation after lung volume reduction surgery

**DOI:** 10.1007/s00464-023-10559-z

**Published:** 2023-11-28

**Authors:** Alberte Lund, Patrick Soldath, Erika Nodin, Henrik Jessen Hansen, Michael Perch, Kristine Jensen, Kåre Hornbech, Anna Kalhauge, Jann Mortensen, René Horsleben Petersen

**Affiliations:** 1 Department of Cardiothoracic surgery, Rigshospitalet, Blegdamsvej 9, 2100 Copenhagen, Denmark; 2 Department of Cardiology, Section for Lung Transplantation and Respiratory Medicine, Rigshospitalet, Copenhagen, Denmark; 3 Department of Diagnostic Radiology, Rigshospitalet, Copenhagen, Denmark; 4 Department of Clinical Physiology and Nuclear Medicine, Copenhagen University Hospital - Rigshospitalet, Copenhagen, Denmark; 5 Department of Clinical Medicine, Faculty of Health and Medical Sciences, University of Copenhagen, Copenhagen, Denmark

**Keywords:** Prolonged air leak, Risk factors, Reoperation, COPD, Lung volume reduction surgery

## Abstract

**Objectives:**

Lung volume reduction surgery (LVRS) has proven an effective treatment for emphysema, by decreasing hyperinflation and improving lung function, activity level and reducing dyspnoea. However, postoperative air leak is an important complication, often leading to reoperation.

Our aim was to analyse reoperations after LVRS and identify potential predictors.

**Methods:**

Consecutive single-centre unilateral VATS LVRS performed from 2017 to 2022 were included. Typically, 3–5 minor resections were made using vascular magazines without buttressing. Data were obtained from an institutional database and analysed. Multivariable logistic regression was used to identify predictors of reoperation. Number and location of injuries were registered.

**Results:**

In total, 191 patients were included, 25 were reoperated (13%). In 21 patients, the indication for reoperation was substantial air leak, 3 patients bleeding and 1 patient empyema. Length of stay (LOS) was 21 (11–33) vs. 5 days (3–11), respectively. Only 3 injuries were in the stapler line, 13 within < 2cm and 15 injuries were in another site. Multivariable logistic regression analysis showed that decreasing DLCO increased risk of reoperation, OR 1.1 (1.03, 1.18, *P* = 0.005). Resections in only one lobe, compared to resections in multiple lobes, were also a risk factor OR 3.10 (1.17, 9.32, *P* = 0.03). Patients undergoing reoperation had significantly increased 30-day mortality, OR 5.52 (1.03, 26.69, *P* = 0.02).

**Conclusions:**

Our incidence of reoperation after LVRS was 13% leading to prolonged LOS and increased 30-day mortality. Low DLCO and resections in a single lobe were significant predictors of reoperation. The air leak was usually not localized in the stapler line.

**Graphical abstract:**

Key question: What characterizes reoperations after lung volume reduction surgeries?

Key findings: Lung injuries were predominantly located away from the original surgical site.

Take home message: Lung injuries remote from the stapler line is frequent during reoperation after lung volume reduction surgery.

Location of lung injuries found during reoperation after lung volume reduction surgery

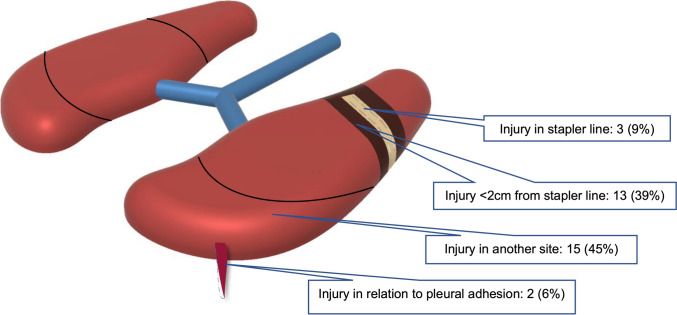

Lung volume reduction surgery (LVRS) is an acknowledged treatment of severe emphysema. It increases walking distance and quality of life [1]. However, air leak continues to be a considerable challenge in the postoperative patient care. Prolonged air leak (PAL) increases length of hospital stay, readmission rate, intensive care unit (ICU) admission and postoperative pneumonia [2].

Since the National Emphysema Treatment Trial (NETT) [3] was carried out more than 20 years ago, LVRS has evolved. More LVRS centres focus on minimally invasive approaches introducing video-assisted thoracoscopic surgery (VATS) instead of median sternotomy or thoracotomy. The quality of paraclinical tools used in patient selection has improved, especially with the single-photon emission computed tomography (SPECT) scans and computed tomography (CT) software, providing a more accurate emphysema assessment in the lung tissue.

PAL is the primary challenge after LVRS, with prolonged PAL (> 7 days) incidence of 24–46% [4].

In NETT, an interim analysis identified a subgroup of patients with high mortality. These patients had Forced Expiratory Volume in 1s (FEV1) < 20% of predicted and either diffusing capacity for carbon monoxide (DLCO) < 20% of predicted or homogenous emphysema. As a consequence, patients with these characteristics have subsequently often been excluded from LVRS. In this fragile patient group, the 30-day mortality rate reported was 16% compared to 2.2% for patients not in the high-risk group. Predictors for PAL were low DLCO, predominant upper lobe disease and extensive pleural adhesions [2].

Our aim in this study was to identify predictors for reoperation and to characterize the intraoperative findings and surgical outcomes.

## Patients and methods

### Patient selection

The study was approved by the Institutional Review Board. Patient informed consent was not required. Conducted as a retrospective cohort study, we included a consecutive series of patients with moderate to severe emphysema eligible for unilateral LVRS from 2017 to 2022. Lung function and medical history were screened according to the inclusion criteria listed in Table 1 before preoperative workup.

**Table 1:** Inclusion criteria for LVRS

Moderate/severe COPD, MRC-score > 2
< 75 years old
17 < BMI > 30
FEV1 > 20% and < 45% of predicted
RV > 200% of predicted
DLCO > 20% of predicted
In ideal medical treatment and having completed rehabilitation
A high-resolution CT < 3 months old should accompany referral
Absence of nodules suspicious of malignancy on CT scan
Absence of significant comorbidity including severe cardiovascular disease
LVEF normal
Absence of pulmonary hypertension, TI-gradient < 40 mmHg
Absence of chronic or frequent lung infections
Smoking abstinence six months prior to surgery
Assessed as sufficiently physically fit to undergo surgery and rehabilitation

*COPD* chronic obstructive pulmonary disease, *MRC* medical research council dyspnoea score, *BMI* Body Mass Index, *FEV1* forced expiratory volume in 1s, *RV* residual volume, *DLCO* diffusion capacity of the lung for carbon monoxide, *LVEF* left ventricle ejection fraction, *TI* tricuspid regurgitation gradient

Eligible patients were submitted to physical evaluation and additional testing at the department. This included medical and surgical history, comorbidity, symptoms, smoking status, cardiology evaluation including echocardiography, blood gas, 6-min-walking test (6MWT), lung function evaluation by spirometry, body plethysmography and DLCO.

Subsequently, all patients were discussed at a multidisciplinary team meeting (MDT), which included a pulmonologist, thoracic surgeon, cardiothoracic radiologist and clinical physiology specialist present. Patient history was presented, high-resolution computed tomography (HRCT) and ventilation perfusion scintigraphy including SPECT/CT imaging were evaluated, and treatment strategy for the patient was planned, including resections on a sublobar level.

### Surgical approach

In 2017, the standardized surgical protocol for LVRS was reformed after a clinical immersion at the Department of Thoracic Surgery, University Hospital Zürich, Switzerland. Depending on suitable target areas on SPECT, several minor wedge resections were preferred in order to diminish tissue stress [5]. With this approach, target areas could be located in more than one lobe, though the traditional horseshoe-shaped resection across the upper lobe was still applied if indicated.

Surgeries were performed using a “standard anterior approach” [6] and only by experienced surgeons. Atraumatic techniques were applied, including only use of peanuts to gently retract the lung. One to six minor resections were made by stapling with vascular tan Tri-Staple™ Technology cartilages without buttressing, followed by water test to visualize possible air leaks. Lastly, two Ch 20 chest drains were inserted and connected to a digital drainage system (Topaz Plus) using 2 cmH_2_0 suction. [7]. If postoperative subcutaneous emphysema developed, an additional third chest drain was considered as an alternative to increasing suction pressure. This was done to prevent enlarging of the fistula or creating further lung injury. Pain management was administered in line with our standard protocol [8].

When the patient was ready for extubation, the double lumen tube was replaced with a laryngeal mask, to prevent coughing and adjoining development of air leaks.

From the first postoperative day, patients were encouraged to physical activity within the physical limitations and with the assistance of a physiotherapist.

The final drain was removed when air leak was < 20 ml/min and fluid loss < 500 ml both for 12 continuous hours, and the patient had been sufficiently mobilized. Two hours after drain removal a chest x-ray was performed.

The indication for reoperation was set by the operating surgeon and established on the basis of a clinical evaluation including appearance of considerable subcutaneous emphysema and the severity of either haemorrhage or air leak. The latter with an approximate cut-off of 1500 ml/min or increasing. For this patient group, reoperation was preferred. Patients with minor air leak for several days were offered blood pleurodesis.

### Statistics

Our primary aim of this study was to review the reoperations performed on this patient group and determine the location of the air leaks. We then compared the surgical outcomes for the reoperated group and the not reoperated group to investigate, whether reoperation constituted a risk. Lastly, we conducted a multivariable logistic regression to isolate predictors for reoperation.

Summary data are presented as mean including standard deviation for parametric data and median including interquartile range for nonparametric data, using appropriate statistical tests.

The categorical postoperative variables were compared individually using the *X*^2^-test to stratify the surgical outcomes for the two groups. Preoperative variables listed in Table 2 were tested with univariable logistic regression with the outcome “reoperation”. Variables with *P* < 0.1 at univariable analysis were included in the multivariable logistic regression. Overall, *P* values < 0.05 were accepted as statistically significant.

**Table 2:** Preoperative patient demographics

Characteristics	All (*n* = 191)
Patients	191
Women, n (%)	112 (59)
Age, years**	65 (59.0, 70.5)
Former BLVR, n (%)	25 (13)
Sputum production limiting activity level, n (%)	31 (16)
> 1 admission with pneumonia last year, n (%)	38 (20)
> 1 admission with exacerbation last year, n (%)	34 (18)
LTOT use, n (%)	14 (7)
CCI**	3 (3, 4)
Smoking pack years**	40 (30.0, 49.5)
A1AD, n (%)	18 (9)
BMI, kg/m2**	23.1 (21.1, 25.9)
Respiratory parameters	
CAT score**	20 (16, 23)
MRC score**	4 (3, 4)
FEV1, %predicted**	28 (22.8, 34.6)
DLCO, %predicted**	35.8 (29.0, 41.8)
FVC, %predicted*	71.1 (17.0)
TLC, %predicted**	134 (129.9, 143.4)
RV/TLC, %*	66.4 (7.31)
6-min walking test	
Walking distance, metres*	309 (104)
Maximum heartrate during test*	117 (16.7)
Minimum blood saturation during test, %**	86 (81, 90)

Data presented as n (%)

*BLVR* bronchial lung volume reduction, *LTOT* long term oxygen treatment, *CCI* charlson comorbidity index, *A1AD* Alpha 1 anti-trypsine deficiancy, *BMI* Body Mass Index, *CAT* COPD assessment test, *MRC* medical research council dyspnoea score, *FEV1* forced expiratory volume in 1s, *DLCO* diffusion capacity of the lung for carbon monoxide, *FVC* forced vital capacity; *TLC* total lung capacity, *RV* residual volume

* Mean (standard deviation)

** Median (interquartile range)

All statistical calculations were carried out using R Studio 2022.07.2 (The R Foundation for Statistical Computing, Vienna, Austria) and performed by the authors alone.

## Results

A total of 191 unilateral LVRS procedures were performed, whereas 21 of these procedures were subsequent contralateral resections. The median age was 65 years (IQR: 59.0–70.5) and 59% were female. Additional preoperative characteristics are listed in Table 2.

Collectively, 25 patients were reoperated within 30 days of the primary procedure (13%). In 21 patients (84%), the indication was substantial air leak. Three patients (12%) were reoperated for bleeding and one patient (4%) for an empyema.

In one patient reoperated for massive air leak, we were not able to localize any lung injuries at all.

**Fig. 1:**
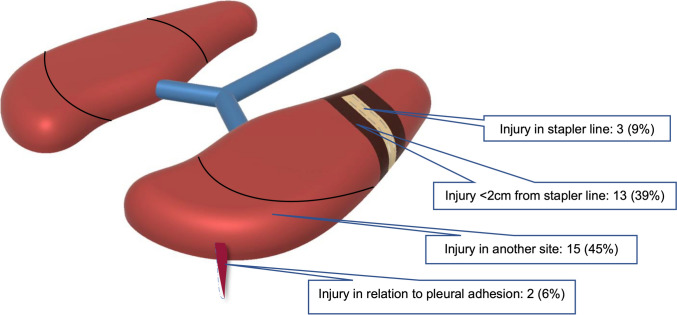
Localization of lung injuries

In total 33 lung injuries were revealed. Eight patients had more than one injury. The location of injuries is depicted in Fig. 1 and Table 3.

**Table 3:** Intraoperative findings during reoperation

Indication	
Substantial air leak	21 (84%)
Bleeding	3 (12%)
Empyema	1 (4%)
Number of lung injuries	
0 injury	5 (20%)
1 injury	12 (48%)
2 injuries	5 (20%)
3 injuries	3 (12%)
Location of injuries	
In stapler line	3 (9%)
< 2cm from stapler line	13 (39%)
other	15 (45%)
In relation to pleural adhesions	2 (6%)
Surgical devices/strategy	
Stapler	20
Pleural tent	10
Dressing (Tachosil + Verizet)	6
Progel	9

The reoperated patients had significantly longer LOS 21 vs. 5 days, more days with chest drain OR 1.11 (95% confidence interval, CI 1.07, 1.17, *P* < 0.001), and PAL OR 5.9 (CI 2.35, 16.94, *P* < 0.001).

The 30-day mortality was significantly increased for the reoperated patients. The risk of ICU admission for the two groups did not differ (Table 4).

**Table 4:** Surgical outcomes for reoperated and not reoperated patients

Postoperative characteristics	Total	Reoperated	Not reoperated	OR	*p* value
Patients	191	25 (13%)	166 (87%)		
Days with chest drain*	4 (2, 11)	16 (9, 23)	3 (2, 9)	1.11 (1.07–1.17)	< 0.001
Length of stay*	6 (3, 14)	21 (11, 33)	5 (3, 11)	1.05 (1.02–1.08)	< 0.001
Prolonged air leak (> 7days)	67 (35%)	18 (72%)	49 (30%)	5.9 (2.35–16.94)	< 0.001
Readmission rate	25 (13%)	3 (16%)	22 (18%)	0.81 (0.19–2.28)	0.91
Postoperative pneumonia	32 (17%)	4 (16%)	28 (17%)	0.94 (0.26–2.70)	0.91
Postoperative empyema	5 (3%)	2 (8%)	3 (2%)	4.72 (0.60–30.00)	0.10
30 days mortality	7 (4%)	3 (12%)	4 (2%)	5.52 (1.03–26.69)	0.02
Admittance to ICU	12(6%)	3 (12%)	9 (5%)	2.38 (0.50–8.69)	0.21

*OR* odds ratio (95% confidence interval)

*Median (interquartile range)

All three patients in the reoperated group died from respiratory failure within few days from the surgery. One was complicated by an empyema. In the not reoperated group, one patient suffered mesenterial ischaemia and respiratory failure and died subsequently. One died from a pressure pneumothorax and the remaining two patients died from respiratory failure.

Multivariable logistic regression analysis showed that decreasing DLCO significantly increased the risk of reoperation by OR 1.1 (CI 1.03, 1.18, *P* = 0.005). Furthermore, resections in only one lobe compared to resections in multiple lobes were a risk factor with OR 3.10 (CI 1.17, 9.32, *P* = 0.03). Number of staplers or resections did not affect the risk of reoperation (Table 5). Risk of reoperation for patients resected in the upper lobe compared to patients resected in non-upper lobes was not significant. Charlson Comorbidity Index (CCI) and COPD Assessment Test (CAT) were significant in the simple logistic regression, however not in the multiple regression model.

**Table 5:** Predictors for reoperation using logistic regression

Characteristics	Univariable		pvalue	Multivariable		pvalue
	OR	CI		OR	CI	
Resection from single lobe vs. Multiple lobes	2.67	1.07–7.69	0.046	3.1	1.17–9.38	0.03
Upper lobe resected vs. Upper lobe NOT resected	1.61	0.66–4.35	0.31			
DLCO (% of predicted, decreasing)	1.08	1.02–1.15	0.003	1.1	1.03–1.18	0.005
CAT score	1.08	1.00–1.16	0.057	1.07	0.99–1.17	0.09
CCI score	1.41	0.94–2.14	0.095	1.37	0.88–2.15	0.17
Number of staplers	0.97	0.84–1.12	0.69			
Number of resections	0.93	0.64–1.31	0.68			

*OR* odds ratio, *CI* 95% confidence interval, *DLCO* diffusion capacity of the lung for carbon monoxide, *CAT* COPD assessment test, *CCI* charlson comorbidity index

## Discussion

The incidence of reoperation after LVRS in this population was 13%, which is higher than in comparable studies. In the NETT reoperation for air leak was 3.3% [9]. Reoperation rates are reported up to 13% [10].

The criteria for reoperation vary among centres. In this study, the operating surgeon decided the indication for reoperation, often resulting in reoperation few days after the primary operation. Other reports have not scheduled reoperations for massive air leak before 15 days if not resolved prior [11].

Hospital LOS was six days in total, with five days and 21 days for the not reoperated and reoperated patients, respectively. This is in line with other studies, where overall LOS was 10–14 days collectively [9]. Expectedly, the reoperated group had significantly longer LOS.

The 30-day mortality rate was 4% in total, which is similar to other studies (2.4%–4%) [12]. However, 30-day mortality was significantly higher in the reoperated group compared to the non-reoperated group (12% vs. 2%).

In this study, PAL > 7days was at 30% and 72% in the non-reoperated and reoperated group, respectively. PAL for LVRS is reported at 46–80% for PAL > 7 days [13]. With these high numbers, PAL is the most common complication after LVRS, causing morbidity and mortality, and increasing medical costs. Reducing PAL presents the most significant challenge in improving the surgical course for LVRS [14]. In this study, we focus on findings during reoperations in an attempt to characterize the air leaks further. We found that only few patients had an air leak in the stapler line (9%) or adjacent to a pleural adhesion (6%). More than 80% of the lung injuries were located away from the surgical site, which indicates that the general pathology of emphysematic lung tissue presents the actual challenge.

Standard approach is to test for air leak before the end of the procedure, and therefore, it is speculated that some of the air leaks might not be present until after the primary surgery, and therefore caused by the physiologic changes in the lung tissue rather than by a specific surgical trauma [11].

Pneumothorax is the primary complication to bronchial lung volume reduction with endobronchial valves (EBV). Recently published data from the CELEB trial showed an incidence of pneumothorax as high as 30.4% [15]. Another study found that 43% of cases were complicated by pneumothorax needing additional treatment for persistent air leak besides chest drain [16].

Peripheral injury occurring after central intervention coincides with our theory, that the underlying lung disease is the problem rather than iatrogenic injuries.

An earlier study concluded that air leak occurred primarily in the proximal stapler line and buttressed staplers with bovine pericardium effectively minimized these leaks [17]. Also, buttressed staplers have proved effectively decreasing days with chest drain; however, LOS and rate of reoperation were unaffected [18]. Furthermore, buttressed staplers have been reported to induce serious complications such as haemoptysis, metalloptysis and interstitial pneumonia [19].

In this study, we found very few lung injuries in the stapler lines. It is hypothesized in other studies, that the staplers redistribute forces across the tissue, creating subsequent tears and air leaks remote from the resection site [20]. In that case, buttressing will not provide additional effect. Our use of Tri staple technology originates from the intention of distributing the tension on the tissue evenly. The choice of vascular staplers originates in the presumption that it will minimize the injuries in this very thin and vulnerable emphysematous tissue. However, scientific verification of this is absent. Mainly because of the intraoperative findings in this study, we do not suspect buttressed stapler will solve the challenge of postoperative airleaks in this patient group. However, in light of the steep reoperation rate and high mortality rate in the reoperated group, we continuously consider all improvements in our procedures to bring down the rate of complications.

Untraditional surgical techniques have been explored to reduce or solve the issue of air leak. Tacconi et al. introduced plication on awake patients using epidural analgesia and had PAL in only 18% of the cases [11]. Results being ambiguous on this subject and pointing to air leaks away from the stapler line, it should be thoroughly considered whether the extra cost of applying buttressed staplers is reasonable.

Our results indicate that resections in only one lobe increase the risk of reoperation. It is hypothesized that spreading the resection to several lobes would minimize the tension on the lung tissue and therefore protect against air leak [4]. But the exact mechanism has yet to be uncovered. It has been suggested by Cooper et al [21]. that a surgical approach, where one continuous line of excision is preferred, over several wedge excisions, prevents air leak. This approach has later in general been changed towards performing several minor resections [5]. We believe our data contribute to this knowledge, with reduction of airleak by distributing resections on multiple lobes.

Only two of the 33 injuries found during reoperation were coinciding with a pleural adherence. As clinical experience indicates that pleural adherences increase risk of postoperative air leak, we expected a substantially greater incidence. Furthermore, pleural adhesions have been found to increase the risk and duration of air leak in the NETT [2]. For endobronchial lung volume reduction, pleural adherences have been identified as a predictor of pneumothorax [22].

Pleural adhesions could possibly still be a risk factor for the patients who were not reoperated. These data from the primary surgery are not available.

Some surgeons prefer a surgical technique where a pleural tent is performed during the primary surgery, to prevent postoperative air leak [23]. A meta-analysis confirmed similar findings for lobectomies [24]. Adversely, the pleural tent procedure has also been tried on patients with primary or secondary pneumothorax, with increasing LOS and days with chest drain as a result [25].

At our centre, pleural tent is not standard of care, merely intermittently used as additional measure against air leak during reoperation.

During the 5-year period of data collection, the indication for pleural tent has gradually changed and is now used more frequently during reoperation, as a method of diminishing air leak. One of the obvious benefits of this procedure is the lack of foreign body involvement.

EBV as treatment for PAL have been evaluated in a review from 2015 [26]. The review included air leak of all causes including surgical complication; however, most air leaks seized within 24 h. These results point to EBV being a possible way of handling PAL in cases, where reoperation is contraindicated or a non-favourable option. The issue of collateral ventilation and bronchoscopic location of the air leak does constitute a challenge [27].

Sealants are often applied as a supplement to staplers in order to prevent air leak after lung surgery. A systematic Cochrane review on effect of sealants in lung surgery uncovered reduced air leak in 12 out of 16 trials; however, it did not translate into reduced LOS [28].

Autologous fibrin sealants could reduce the incidence of PAL and duration of chest drainage after LVRS [29, 30]. However, from a cost/benefit perspective, postoperative autologous blood patch pleurodesis could be a more sensible choice.

A review from 2021 showed effectiveness of postoperative autologous blood pleurodesis on treating air leak within 48 h for 85.7% of patients with postoperative air leak [31], however not focussing solely on LVRS patients. It has not been explored whether this treatment is effective and safe in a LVRS population. We do not find the scientific evidence for the effect of chemical pleurodesis sufficient [32], and furthermore, we are reluctant to use chemical because the concomitant pleural adherences can cause surgical challenges related to potential later lung transplantation.

We found that low DLCO and resections in only one lobe instead of several lobes were predictors of reoperation. Isolated upper lobe resection showed no effect on reoperation rate in our study.

In the NETT, PAL was associated with low DLCO, pleural adhesions, predominantly upper lobe disease, steroid usage and Caucasian race. Mortality did not differ for the PAL group; however, rate of postoperative pneumonia and admission to the intensive care unit were increased [2, 3].

The NETT group did specify a high-risk patient group with DLCO < 20% or FEV1 < 20% with increased mortality risk [3]. As a consequence, several centres have incorporated a DLCO < 20% as an exclusion criterion for surgery. However, improvement of lung function following LVRS has even been proved beyond the NETT criteria for DLCO down to 15% without increased mortality [3, 33]. Pursuing to minimize the risk of reoperation in our centre, increasing the DLCO cut-off is a possibility.

The retrospective nature of this study presents an obvious limitation. The small sample size and the indication for reoperation set by the individual surgeon also present a limitation, though we have no evidence to support expectations of a different outcome with a more conservative or aggressive approach. Not many manuscripts have been published on complications after LVRS despite the massive challenge of PAL. The data presented in this manuscript provide insights into this challenge.

## Conclusion

Air leak is the predominant reason for reoperation in LVRS and is associated with increased LOS and mortality. Minimizing air leak therefore remains the main challenge in LVRS.

During reoperations, we found injuries predominantly occurred away from the stapler line. This contributes to current knowledge of understanding the pathology of postoperative air leak.

Predictors for reoperation were low DLCO and resections in only one lobe, suggesting that multiple minor resections in several lobes are preferred. Indication for LVRS should be carefully evaluated in patients with low DLCO.

## Abbreviations

A1AD: Alpha 1 anti-trypsin deficiency
BMI: Body Mass Index
BLVR: Bronchial lung volume reduction
CAT: COPD assessment test
CCI: Charlson comorbidity index
COPD: Chronic obstructive pulmonary disease
CT: Computed tomography
DLCO: Diffusion capacity of the lung for carbon monoxide
EBV: Endobronchial valves
FEV1: Forced expiratory volume in one second
FVC: Forced vital capacity
HRCT: High-resolution computed tomography
LOS: Length of stay
LTOT: Long-term oxygen treatment
LVEF: Left ventricle ejection fraction
LVRS: Lung volume reduction surgery
MRC: Medical research council dyspnoea score
NETT: Nation emphysema treatment trial
PAL: Prolonged air leak
RV: Residual volume
SPECT: Single-photon emission computed tomography
TLC: Total lung capacity
RV: Residual volume
VATS: Video-assisted thoracoscopic surgery
6MWT: Six-minute walking test
